# Dissecting aneurysm of sinus of Valsalva into interventricular septum and rupturing into left ventricle through multiple sinuses: a rare case report

**DOI:** 10.1093/ehjcr/ytae417

**Published:** 2024-08-20

**Authors:** Shubham Sharma, Jayal Shah, Riyaz Charaniya, Rakesh Dash

**Affiliations:** Department of Cardiology, U. N. Mehta Institute of Cardiology and Research Centre (UNMICRC), Civil Hospital Campus, Asarwa, Ahmedabad 380016, Gujarat, India; Department of Cardiology, U. N. Mehta Institute of Cardiology and Research Centre (UNMICRC), Civil Hospital Campus, Asarwa, Ahmedabad 380016, Gujarat, India; Department of Cardiology, U. N. Mehta Institute of Cardiology and Research Centre (UNMICRC), Civil Hospital Campus, Asarwa, Ahmedabad 380016, Gujarat, India; Department of Cardiology, U. N. Mehta Institute of Cardiology and Research Centre (UNMICRC), Civil Hospital Campus, Asarwa, Ahmedabad 380016, Gujarat, India

**Keywords:** Sinus of Valsalva aneurysm (SOVA), Dissecting aneurysm of the interventricular septum (DAIS), Ruptured sinus of Valsalva (RSOV), Case report

## Abstract

**Background:**

Ruptured sinus of Valsalva (RSOV) is a rare disorder, which usually involves the right coronary sinus (RCS) or the non-coronary sinus (NCS) and ruptures usually into the right-sided chambers. Involvement of the left coronary sinus (LCS) and multiple sinuses, rupture into the left ventricle (LV), and dissecting aneurysm of the interventricular septum (IVS) have all been scarcely reported.

**Case summary:**

A 24-year-old male presented with complaints of exertional fatigue, palpitations, and chest pain with signs of aortic run-off like wide pulse pressure, collapsing pulse along with cardiomegaly, and a diastolic murmur. Echocardiography revealed sinus of Valsalva aneurysms (SOVAs) involving both the RCS and LCS with RCS aneurysm dissecting the IVS and rupturing into the LV and another multilobulated aneurysm from LCS rupturing into the LV. Findings were confirmed on computed tomography (CT) aortogram, and the patient underwent successful surgical repair.

**Discussion:**

Sinus of Valsalva aneurysm is a rare disorder which usually ruptures into the right-sided chambers. The involvement of multiple sinuses and rupture into the IVS is extremely rare (<2%). Aneurysm dissecting the IVS can lead to complete heart block (CHB) and sudden death. Involvement of the LCS is reported in <5% cases of RSOV, and rupture of such an aneurysm into the pericardial space may lead to cardiac tamponade and can also lead to sudden death. Clinical examination, electrocardiogram, chest X-ray, 2D echocardiography, and CT all help in the diagnosis. Treatment involves surgical repair of the defect.

**Learning points**

- Sinus of Valsalva aneurysm (SOVA) dissecting into the interventricular septum (IVS) and opening in the left ventricle (LV) is a rare complication which can lead to complete heart block and sudden death.
- Aneurysm involving the left coronary sinus can be sometimes life-threatening if it ruptures into the pericardial space leading to acute development of cardiac tamponade.
- Ruptured sinus of Valsalva with involvement of multiple sinuses has rarely been reported.
- Clinical findings of aortic run-off like collapsing pulse, wide pulse pressure, cardiomegaly, and a diastolic murmur help in clinching the diagnosis of RSOV into the LV.
- Confirmation of diagnosis may require multimodality imaging including chest X-ray, 2D echocardiography, and computed tomography aortogram to define the extent of involvement and possible complications.
- Timely surgery can be life-saving.

## Introduction

Congenital sinus of Valsalva aneurysm (SOVA) results from the separation between aortic media and annulus fibrosus as a result of weakened elastic lamina.^1–3^ The Valsalva aneurysm usually involves the right coronary sinus (RCS) or the non-coronary sinus (NCS) and less commonly left coronary sinus (LCS). It usually ruptures into a right-sided chamber, and rupture into the left ventricle (LV) is rare. Dissecting aneurysm into interventricular septum (IVS) has been scarcely reported. Involvement of multiple sinuses and multilobulated aneurysms are also uncommon. Here, we report first such case who had all the rare findings mentioned above. The patient underwent successful surgical repair of the defect.

## Summary figure

**Figure:**
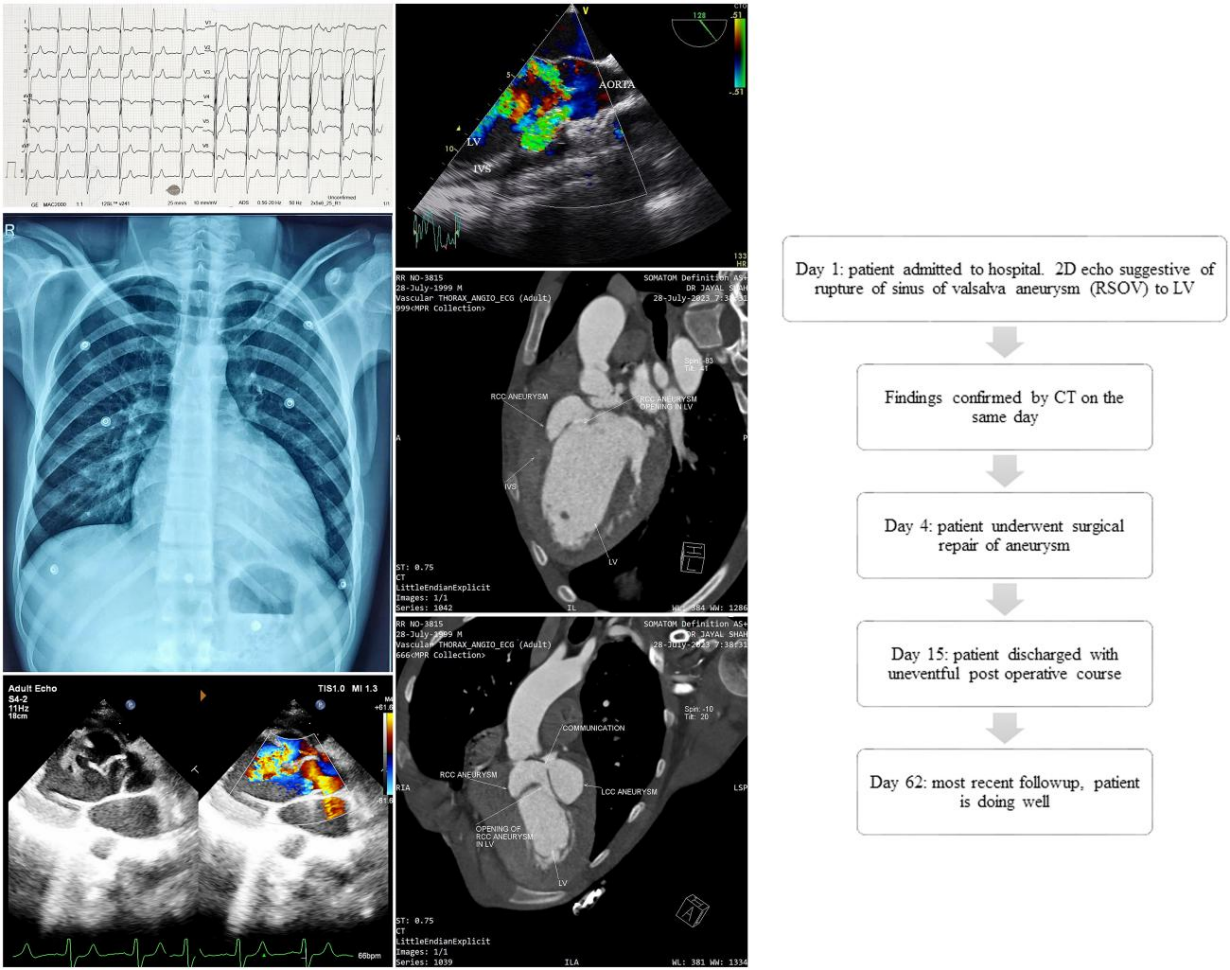


## Case presentation

A 24-year-old male with no significant past history presented with complaints of fatigue and exertional palpitations since 6 months and chest pain 8 days back. Peripheral pulses were bounding and collapsing in character (water hammer pulse), and prominent carotid pulsations were visible (Corrigan’s sign). Systolic blood pressure of 110 mmHg and diastolic blood pressure of 40 mmHg were recorded in both the upper limbs. Systolic blood pressure was 166 mmHg in both lower limbs. Cardiac apex was shifted to the 6th intercostal space, 2 cm lateral to the mid-clavicular line, hyper-dynamic in character. Prominent diastolic thrill was palpable in the left parasternal area. A high-pitched loud pan diastolic murmur was audible all over the precordium, best heard over the left parasternal area in sitting and leaning forward position.

**Figure 1:**
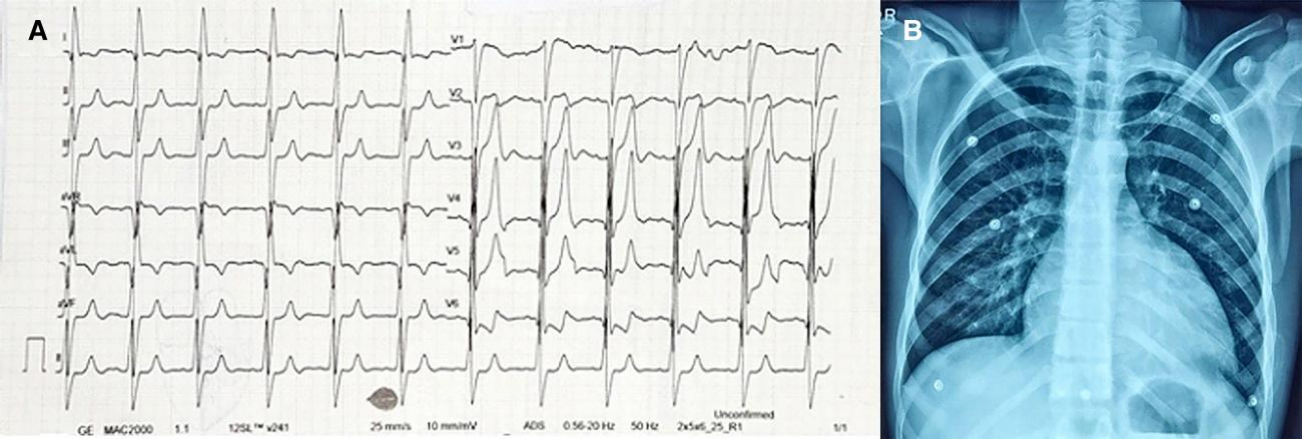
(*A*) Electrocardiogram showing features of left ventricle volume overload. (*B*) Chest X-ray showing cardiomegaly with left ventricle type of apex.

Electrocardiogram (*Figure 1A*) had left bundle branch block (LBBB), left-axis deviation (LAD), LV enlargement with Q waves in lateral leads (I, avL, v5, v6), suggestive of LV volume overload. Chest X-ray had cardiomegaly with LV type of apex (*Figure 1B*).

**Figure 2:**
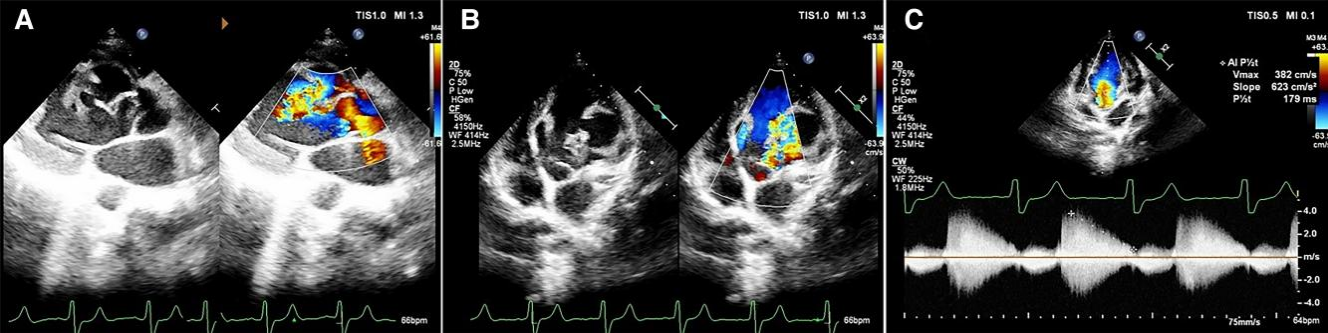
(*A–C*) Transthoracic echocardiogram. (*A*) Parasternal long-axis view with colour Doppler showing ruptured sinus of Valsalva from the right coronary sinus into the interventricular septum and opening into the left ventricle. (*B*) Apical five-chamber view with colour Doppler showing two separate sinus of Valsalva aneurysms from the right and left coronary sinuses rupturing into the left ventricle. (*C*) Continuous wave Doppler at the site of rupture of aneurysm showing diastolic flow into the left ventricle with pressure half time of 179 ms suggestive of severe aortic regurgitation.

2D echocardiography (*Figure 2*) revealed dilated LV, severe LV systolic dysfunction with ejection fraction of 20%, and global hypokinesia. In parasternal long-axis view, a jet of blood could be appreciated, arising from the RCS, dissecting into a tract within the IVS and entering into the LV. On parasternal short-axis view, a multilobulated aneurysmal sac was visualized impinging on the lateral wall of the LV extending from the base to the mid-LV and opening into the LV. The SOVA originating from LCS seemed to be multilobulated. It divided into two aneurysmal outpouchings that were seen to be communicating with each other and opened into the LV through separate openings. Continuous wave Doppler revealed regurgitant diastolic jet from the aorta to LV through the RSOV.

**Figure 3:**
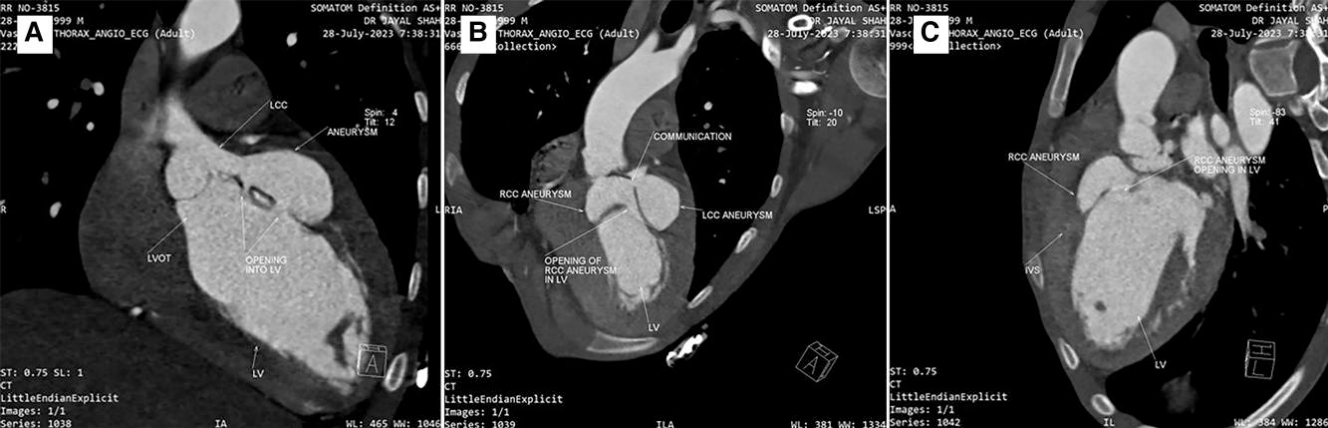
(*A*) Computed tomography showing aneurysm originating from the left coronary sinus and rupturing into the left ventricle through two separate openings. (*B*) Image showing the two aneurysms from the right and left coronary sinuses communicating with each other and the right sinus of Valsalva aneurysms rupturing into the left ventricle. (*C*) Image showing the sinus of Valsalva aneurysms from the right coronary sinus rupturing into the interventricular septum and opening into the left ventricle.

Computed tomography (CT) aortogram (*Figure 3A–C*) revealed aneurysm measuring 32.2 × 22.5 × 13.7 mm, arising from the RCS protruding into the IVS and communicating with the LV. Another multilobulated aneurysm was noted arising from the LCS (23.2 ×42.6 × 58.7 mm) and communicating with the LV (see Supplementary material).

**Figure 4:**
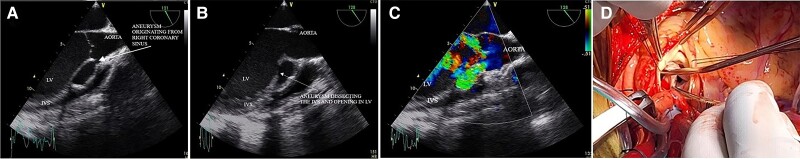
(*A–C*) Intra-operative transoesophageal echocardiogram showing ruptured sinus of Valsalva from the right coronary sinus dissecting the interventricular septum and opening into the left ventricle. (*D*) Ruptured sinus of Valsalva being repaired surgically by patch closure.

Intra-operative transoesophageal echocardiogram (TEE) confirmed SOVA from RCS dissecting into IVS and opening into the LV (*Figure 4A–C*). During surgery, the two separate SOVAs were visualized, and the patient underwent successful patch closure of both through the aortic root via an aortotomy. Post-operative course was uneventful, and 2D echo had no residual flow into the LV (see Supplementary material). On the most recent follow-up (62 days), the patient was symptomatically better.

## Discussion

Sinus of Valsalva is a dilatation arising from the aortic sinus and is named in relation to the respective valve cusp. Aneurysm of the sinus is a rare disorder which can be congenital as well as acquired (uncommon). Its prevalence is estimated to be 0.09% in the general population with male-to-female ratio of 4:1 and is more common in Asians.^1,2^ Congenital SOVA results from the separation between the aortic media and annulus fibrosus as a result of weakened elastic lamina.^3^ Congenital aneurysms are associated with bicuspid aortic valve, ventricular septal defects, coronary artery anomalies, and aortic regurgitation (AR). Causes of acquired aneurysms include trauma, tuberculosis, syphilis, infective endocarditis, Behçet disease, and atherosclerosis.^1^

Clinical presentation in a patient with RSOV aneurysm depends on the rate of rupture, size, and sinus involved. Aneurysms which rupture acutely may present with sudden chest pain or dyspnoea followed by progressive symptoms of heart failure. However, such a classical presentation is seen in only one-third of patients. Sometimes a small rupture enlarges over time leading to gradually progressive symptoms of volume overload. Sudden death has been reported in cases of aneurysm rupturing into the pericardium and IVS leading to CHB.

Aneurysm of the RCS usually ruptures into the right ventricle and that of the NCS into the right atrium. Sinus of Valsalva aneurysm usually involves the RCS (80%) and NCS (16%), while LCS involvement is uncommon (<5%).^4^ Involvement of more than one sinus and rupture into IVS (<2%) is extremely rare.

A study by Yang *et al.*^5^ found the involvement of LCS in 0.7%, multiple sinuses involvement in 2.6%, and SOVA rupturing into the LV in 1.5% of all patients with ROSV. Modified Sakakibara classification system for RSOV classifies it into five different types based on the site of rupture.^6^

Warthen in 1947 was the first to report a SOVA dissecting into the IVS.^7^ Such a SOVA usually arises from the right sinus that lies near the IVS and is primarily congenital in origin. This aneurysm after gradual enlargement can rupture into any cardiac chamber, and sudden rupture into the IVS can lead to CHB and sudden death. Similarly, SOVA originating from LCS may rupture into the pericardial space and lead to cardiac tamponade and sudden death.

A case report of two patients presenting with SOVA involving the LCS and the RCS dissecting into the IVS and rupturing in LV has been reported by Jain *et al*.^8^ However, this is the first case in which two sinuses were involved in the same patient with one SOVA originating from the RCS, rupturing into the LV after dissecting the IVS and another multilobulated SOVA originating from the LCS and rupturing into the LV through multiple openings.

Clinical suspicion along with multimodality imaging using transthoracic echocardiogram, TEE, and CT aortogram is helpful in diagnosing RSOV and in the pre-operative planning. Cardiac MRI is considered the gold standard for diagnosis. Differential diagnosis includes causes of AR like infective endocarditis of the aortic valve, blunt chest trauma, aortic aneurysm, and aortic dissection, coronary–cavernous fistula, IVS abscess and are also close mimickers. Definitive treatment involves surgical repair.

## Lead author biography


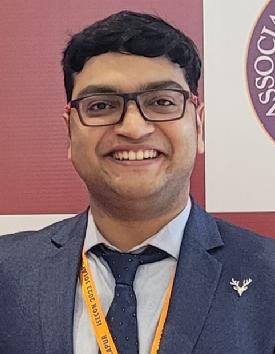


Dr Shubham Sharma is a third year resident pursuing Doctorate of Medicine in cardiology at U N Mehta Institute of Cardiology and Research Centre, Ahmedabad, Gujarat, India

## Supplementary Material

ytae417_Supplementary_Data

## Data Availability

The authors confirm that the data supporting the findings of this case report are available within the manuscript and its online Supplementary material. Further details can be requested by contacting the corresponding author.

## Notes

**Consent:** Written consent for the submission and publication of this case report including images and associated text has been obtained from the patient in line with the COPE guidelines.

**Funding:** This work received no specific grant from any funding agency, commercial or, not-for-profit sectors.

## References

[1] Weinreich M , Yu PJ, Trost B. Sinus of Valsalva aneurysms: review of the literature and an update on management. Clin Cardiol 2015;38:185–189. 25757442 10.1002/clc.22359 PMC6711005

[2] Chu SH , Hung CR, How SS, Chang H, Wang SS, Tsai CH, et al Ruptured aneurysms of the sinus of Valsalva in oriental patients. J Thorac Cardiovasc Surg 1990;99:288–298. 2299866

[3] Edwards JE , Burchell HB. The pathological anatomy of deficiencies between the aortic root and the heart, including aortic sinus aneurysms. Thorax 1957;12:125–139. 13442955 10.1136/thx.12.2.125 PMC1019237

[4] Ott DA . Aneurysm of the sinus of Valsalva. Semin Thorac Cardiovasc Surg Pediatr Card Surg Annu 2006;9:165–176. 10.1053/j.pcsu.2006.02.014 16638563

[5] Yang Y , Zhang L, Wang X, Lü Q, He L, Wang J, et al Correction: echocardiographic diagnosis of rare pathological patterns of sinus of Valsalva aneurysm. PLoS One 2018;13:e0197723. 29768514 10.1371/journal.pone.0197723 PMC5955541

[6] Xin-Jin L , Xuan L, Bo P, Hong-Wei G, Wei W, Shou-Jun L, et al Modified Sakakibara classification system for ruptured sinus of Valsalva aneurysm. J Thorac Cardiovasc Surg 2013 Oct;146:874–878. 23312973 10.1016/j.jtcvs.2012.12.059

[7] Warthen RO . Congenital aneurysm of the right anterior sinus of Valsalva (interventricular aneurysm) with spontaneous rupture into the left ventricle. Am Heart J 1949;37:975–981. 18119903 10.1016/s0002-8703(49)90947-3

[8] Jain A , Achuthan G. Rupture of sinus of Valsalva aneurysm into interventricular septum: role of cardiac CT. Cureus 2019;11:e5589. 31696007 10.7759/cureus.5589 PMC6820894

