# Supplementary figures and images for: Dissecting aneurysm of sinus of Valsalva into interventricular septum and rupturing into left ventricle through multiple sinuses: a rare case report
Source: Eur Heart J Case Rep. 2024 Aug 20;8(8):ytae417. doi: 10.1093/ehjcr/ytae417 (PMC11350371; doi:10.1093/ehjcr/ytae417)

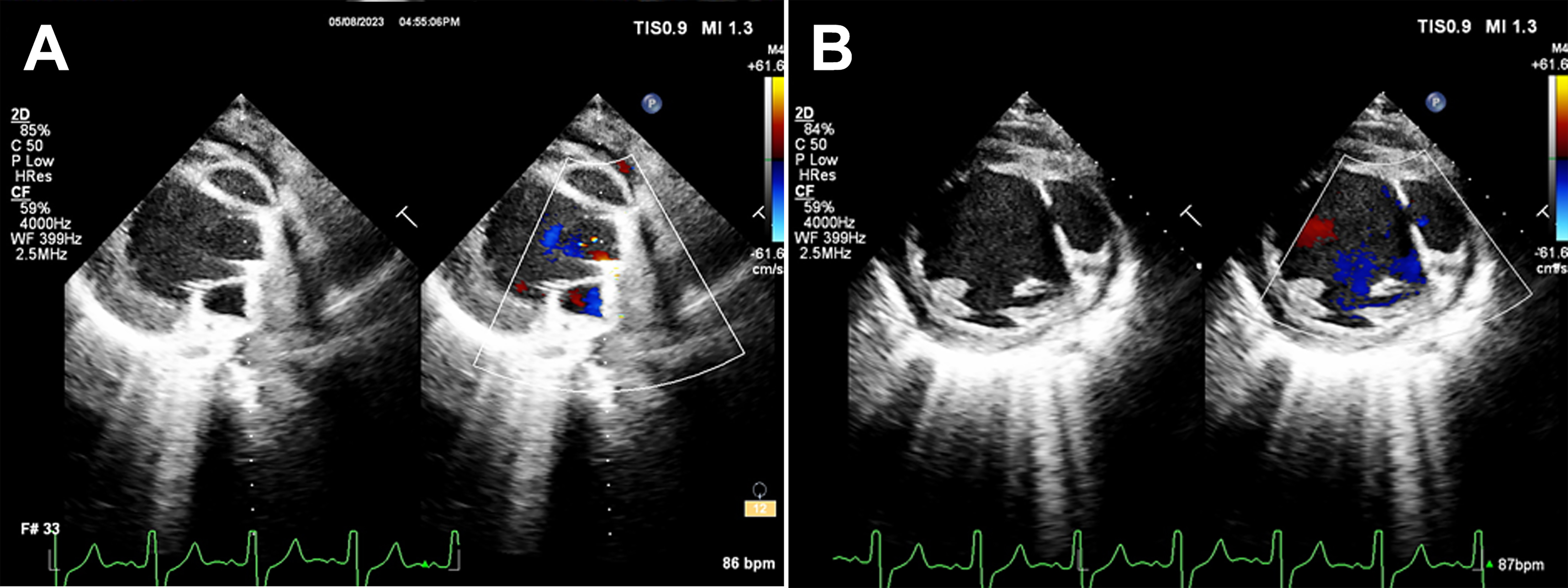

Supplement: ytae417_Supplementary_Data [file ytae417_supplementary_data.zip › POST OP ECHO.tiff]
